# Prevalence of hypertension in Ethiopia: a systematic meta-analysis

**DOI:** 10.1186/s40985-015-0014-z

**Published:** 2015-12-09

**Authors:** Kelemu Tilahun Kibret, Yonatan Moges Mesfin

**Affiliations:** 1 Department of Public Health, Wollega University, Nekemte, Ethiopia; 2 Department of Public Health, Haramaya University, Harar, Ethiopia

**Keywords:** Hypertension, Ethiopia, Prevalence, Blood pressure, Systolic, Diastolic

## Abstract

**Background:**

Hypertension has been increasing in developing countries including Ethiopia. Other than smaller studies, there is no national prevalence study on hypertension in Ethiopia. These smaller studies reported varied prevalence of hypertension. This study is intended to summarize and pool the results of smaller region based studies to provide a national level estimate of the prevalence of hypertension.

**Methods:**

The studies were identified through internet search using the data base of MEDLINE/PubMed, Google scholar, EMBASE, HINARI, Cochrane library and reference lists of previous prevalence studies. We also made manual searches to identify relevant articles. Descriptive information for the original studies is presented in a table and the quantitative results were presented in forest plots. The Cochrane Q test and I^2^ test statistic were used to test heterogeneity across studies. The Pooled estimate of prevalence of hypertension was computed by a random effects model.

**Results:**

One hundred eight titles were identified through electronic searching using keywords. Of these, nine studies were meet the inclusion criteria. A random effect meta-analysis of the results from these 9 studies was conducted to provide an estimate of the prevalence of hypertension in the Ethiopian population. The analysis showed that the prevalence of hypertension among Ethiopian population was estimated to be 19.6 % (95 % CI: 13.7 %, 25.5 %). Subgroup analyses indicated that the prevalence of hypertension is higher in the urban population (23.7 %) than rural and urban combined (14.7 %). The prevalence of hypertension among males (20.6 %) and females (19.2 %) was similar.

**Conclusion:**

This study found that the prevalence of hypertension in Ethiopia is increasing. This evidence suggests that attention has to be given to primary prevention of hypertension in the Ethiopian adult population, especially in the urban population by integrating it with health extension programs.

## Background

Hypertension is a condition in which the blood pressure in arteries or veins is abnormally high and defined as a systolic blood pressure equal to or above 140 mm Hg and/or diastolic blood pressure equal to or above 90 mm Hg [1]. Non-communicable diseases (NCDs) are the major cause of death in the world, accounting for more than 36 million (63 %) of the 57 million deaths that happened in 2008. Almost half, (48 %) of NCD deaths are due to cardiovascular diseases (CVD) [2]. Hypertension is one of the main modifiable risk factors for CVD [3].

The prevalence of raised blood pressure in adults older than 25 years of age was about 40 % in 2008 [4] and contributed to 12.8 % of the total deaths in the world [5]. The World Health Organization (WHO) estimated that around 62 % of CVDs and 49 % of ischemic heart diseases are attributable to high blood pressure in the world [6].

Hypertension is the 4^th^ contributor to premature deaths in developed countries and the 7^th^ in developing countries [7]. Until now, communicable diseases, maternal, and nutritional causes were responsible for the highest burden of morbidity and mortality in Africa [8]. Most recently it has been shifting towards non-communicable diseases and developing countries are facing what is known as “double burden of diseases” [9]. Similarly, in the first half of the twentieth century, high blood pressure was almost non-existent in African societies, but currently estimates show that in some settings in Africa more than 40 percent of adults have hypertension [1, 10].

Although hypertension is a preventable and modifiable risk factor of CVD, the prevention and control of hypertension has not yet received due attention in many developing countries [5, 10]. Ethiopia is one of the lower income countries that has affected by double burden diseases. The WHO 2011 report showed that 34 % of all deaths in Ethiopia were due to NCDs, from which CVD contributes 15 % [2]. A burial surveillance in Addis Ababa also revealed that 51 % of all deaths were due to NCDs, of which CVD was a main cause of death (24 %), and hypertension was responsible for 12 % of the CVD deaths [11]. Another cross-sectional study in South West Ethiopia reported that hypertension contributed 30.9 % of cardiac cases [12]. Different studies in Ethiopia revealed that increased risk of hypertension was associated with older age (> = 45 years), obesity [13], smoking [14] and chat chewing [14, 15].

An up-to-date and comprehensive assessment of the evidence concerning hypertension in Ethiopia is lacking. One community-based cross sectional study done in Addis Ababa showed that the age adjusted prevalence of high blood pressure was 31.5 % among males and 28.9 % among females [16].

Other than reginal studies done in different part of the country, there is no national prevalence study on hypertension in Ethiopia. The prevalence of hypertension also varied largely across these small studies [16–18]. The aim of this study was to estimate the prevalence of hypertension at national level by using the results from these smaller reginal studies. Given the fact that hypertension has been rising in developing countries including Ethiopia, this meta-analysis is designed to consolidate the available data to determine the current magnitude of hypertension among the Ethiopian population.

## Methods

### Study design and search strategy/data source

A systematic meta-analysis was done using published and unpublished articles on prevalence of hypertension in Ethiopia. The studies were found through internet searches using database of MEDLINE/PubMed, Google scholar, EMBASE, HINARI, Cochrane Library and reference lists of previous prevalence studies. The search was done using the following keywords individually or in combination: hypertension, Ethiopia, prevalence, blood pressure, systolic, diastolic. The limit for language was English and the limit for study category was human. The searching of articles was carried out from June, 2014 to November, 2014.

### Study selection

Studies were selected for the meta-analysis if they were conducted in Ethiopia and reported the prevalence of hypertension. After preliminary screening of all the titles obtained from our searches, all abstracts were then assessed for eligibility by two independent researchers based on the inclusion criteria. Disagreement between the two researchers was resolved through discussion and consensus. Finally, studies that met all of the following criteria were included in the meta-analysis: 1) cross-sectional study; 2) conducted in age group of 15 years and above and done in Ethiopia; 3) for classification of hypertension, studies that used the cut-off value of ≥140 mmHg and ≥ 90 mmHg for systolic and diastolic blood pressure respectively or studies that used self-reported use of antihypertensive medication. Studies that used blood pressure measurement on a single visit were also considered; 4) having response rate of ≥80 % (it is the proportion of study participants who gave appropriate response to the study among the sample); 5) reported prevalence of hypertension; 6) the studies that contained original data; 7) Random sampling from a defined population or studies involving entire populations; 8) studies that reported quality assurance methods.

The reviews, letters to editors, case series and case-control studies were excluded from analysis because of insufficient data.

### Data extraction/abstraction

The data extraction was done by two researchers (KT, YM) using a standardized and pretested format. Data extraction included: title, first author, publication year, year of survey, design of the study, study base (population-based or hospital-based), settings (urban, rural), sample size, data collection procedure, age group of study participants, response rate, region of study (study site in the country), sampling methods, definition(s) used for hypertension, prevalence of hypertension (age adjusted or unadjusted if stated). The overall prevalence rate as well as prevalence of hypertension by sex and residency subcategories was also extracted. Disagreements on data extractions between the two investigators were solved by discussion and consensus.

### Operationalization of outcome measures

The primary outcome measure was the percentage of individuals having systolic blood pressure ≥140 mmHg and/or diastolic blood pressure ≥ 90 mmHg. The prevalence of hypertension was calculated by dividing the number of individuals with systolic blood pressure ≥140 mmHg and/or diastolic blood pressure ≥ 90 mmHg by the total number of study subjects (sample), and multiplied by 100.

### Quality assessment

Reporting of response rate greater than or equal to 80 %, appraisal of internal validity of study results, appropriate sampling methods, clear data collection methods and procedures, reported quality assurance methods (training of data collectors, pretesting, and supervision) and representative sample size were considered as study quality indicators. All assessments were entered into preformatted and standardized data extraction forms. Studies were evaluated for quality by using these indicators; those with medium (fulfilling 50 % of quality assessment criteria) and high quality were included for analysis. High quality studies were studies that reported all the above stated points.

### Synthesis of results/statistical analysis

The data entry and analysis was done using Epi data version 3.1 and STATA version 11.0 (STATA Corporation, College Station Texas) software respectively. The original articles were described using forest plot and table. Since there was heterogeneity among studies, random effect model was used to compute the pooled prevalence of hypertension. Random effect model is more conservative than fixed effect model and takes into account any heterogeneity inherent in the meta-analysis. The estimated pooled prevalence rate with its 95 % confidence interval (CI) was presented.

### Sub-group analyses

Sub-group analyses were performed for residency (urban and rural/urban) and sex (male and female).

### Heterogeneity and publication bias

Statistical heterogeneity was evaluated by Cochran’s Q test, which shows the amount of heterogeneity between studies and I^2^ statistic. The I^2^ provides an estimate of the percentage of the variability in effect estimates that is due to heterogeneity rather than sampling error or chance differences. So, the existence of heterogeneity was verified using Cochran’s Q test (*P* < 0.10 indicates statistically significant heterogeneity) and I^2^ test that measures level of statistical heterogeneity between studies (values of 25 %, 50 % and 75 % are to mean low, medium and high heterogeneity respectively).

The Egger weighted regression and Begg rank correlation test methods were used to statistically assess publication bias (*P* < 0.05 was consider as suggestive of statistically significant publication bias).

## Results

### Identified studies

**Fig. 1:**
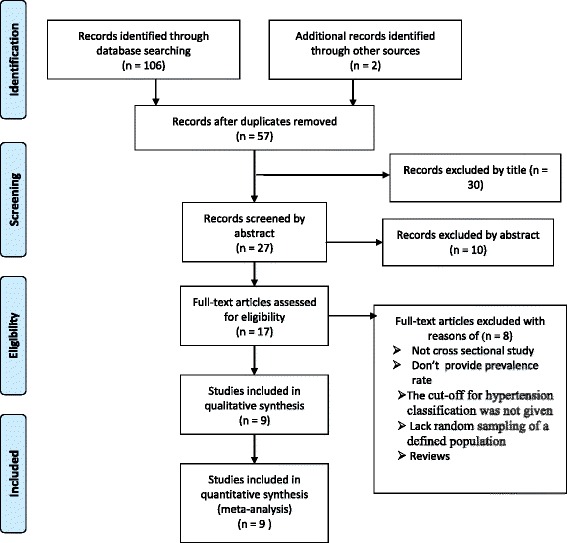
Flow chart diagram describing selection of studies for a systematic review and meta-analysis of prevalence of hypertension in Ethiopia, 2014 (identification, screening, eligible and included studies). Articles may have been excluded for more than one reason

We identified 108 titles by the electronic search in MEDLINE/PubMed, Google scholar, EMBASE, HINARI, Cochrane Library and reference lists of previous prevalence studies. Of which 99 were excluded (51 due to duplication, 30 through review of titles, 10 by reviewing of abstracts, and 8 full text articles due to inclusion criteria). Finally, nine studies were found to be eligible and included in the meta–analysis (Fig. 1).

### Study characteristics

A total of nine studies were included in the meta-analysis. Seven of them were population based [3, 16–21] and two were hospital/institution based [22, 23]. All are cross sectional studies which had study population varied from 395 in Sidama [23] to 3223 in South West Ethiopia of Gilgel Gibe [17], and were conducted between the year 2009 and 2014. These studies represented five regions of country: (Amhara [3], Oromia [18, 22], Southern Region [17, 21, 23], Tigray [20] and Addis Ababa [16, 19] (Table 1). Eight studies described the prevalence of hypertension in both sexes as presented in Table 2.

**Table 1:** Descriptive summary of the nine studies on prevalence of hypertension in Ethiopia and included in the meta-analysis, 2014

Author (Publication year)	Region	Settings (urban/rural)	Study base (population based/hospital-based)	Age group of subjects	Sample size	Response rates (%)	Prevalence with its 95 % CI
Awoke A et al. [3] 2012	Gondar/Amhara	Urban	Population	> = 35	679	97.6	28.3 (24.9–31.7)
Tesfaye F et al. [16] 2009	Addis Ababa	Urban	Population	25–64	3273	88.1	30.3 (28.7–31.9)
Bonsa F et al. [18] 2014	Bedele/Oromia	Urban	Population	> = 15	396	93.8	16.9 (13.2–20.6)
Gudina EK et al. [22] 2013	Jimma/oromia	Urban/rural 57 % from rural	Hospital based	> = 15	774	94.83	13.2 (10.8–15.6)
Nshisso LD et al. [19] 2012	Addis Abba	Urban	Gov’t employee	>15	2153	100	19.1 (17.1–20.8)
Giday A et al. [23] 2010	Sidama zone/Southern Region	Both Urban & rural	Hospital/Population	15-80	444/395	88.9	18.8 (15.1–23.1)
Mengistu MD et al. [20] 2014	Humera/Mekele	Both Urban & rural	Community	> = 18	1183	100	18.1 (15.9–20.3)
Muluneh AT et al. [17] 2012	SW Ethiopia, Gilgel Gibe/Southern Region	Both Urban & rural	Community/population	15–64	3223	81.3	9.3 (8.3,10.3)
Helelo TP et al. [21] 2013	Durame/Southern region	Urban	Population	> = 31	518	98.6	22.4 (18.8,26)

**Table 2:** Descriptive summary of the eight studies on prevalence of hypertension in Ethiopia according to sex of the subjects included in the meta-analysis, 2014

Author (year of Publication)	Region in the Country	Settings (urban/rural)	Age of subjects	Sample size	No. of males	No. of females	Prevalence of hypertension with its CI among	
							Males	Females
Awoke A et al. [3] 2012	Gondar/Amhara	Urban	> = 35	679	323	356	26 (21.4–31)	30.3 (25.6–35.3
Tesfaye F et al. [16] 2009	Addis Ababa	Urban	25–64	3273	1538	2175	31.5 (29–33.9)	28.9 (26.8–30.9)
Bonsa F et al. [18] 2014	Bedele/Oromia	Urban	> = 15	396	267	129	13.1 (9.6–17.7)	24.8 (18.2–32.9)
Gudina EK et al. [22] 2013	Jimma/oromia	Urban/rural 57 % from rural	> = 15	774	308	426	15.3 (11.7–19.7)	11.7 (9.0–15.1)
Nshisso LD et al. [19] 2012	Addis Abba	Urban	>15	2,153	1298	855	22 (20.2–23.8)	14.9 (13.4–16.4)
Giday A et al. [23] 2010	Sidama zone/Southern Region	Both Urban & rural	15-80	395	–	–	– –	–
Mengistu MD et al. [20] 2014	Humera/Mekele	Both Urban & rural	> = 18	1183	443	740	21 (17.5–25.0)	16.4 (13.9–19.2)
Muluneh AT et al. [17] 2012	SW Ethiopia,Gilgel gibe/Southern Region	Both Urban & rural	15-64	3223	1541	1682	10.3 (8.8–11.9)	8.5 (7.3–9.9)
Helelo TP et al. [21] 2013	Durame/Southern Region	Urban	> = 31	518	229	289	26.2 (20.9–32.3)	19.4 (15–24)

### Heterogeneity and publication bias

The included articles exhibited high heterogeneity according to Cochrane Q test (Q test *p* = 0.001) and I^2^ test (I^2^ = 98.6 %), which is indicative to using random effects model. But the Egger weighted regression statistics (*p* = 0.12) and Begg rank correlation statistics (*p* = 0.6) indicated no evidence of publication bias.

### Meta-analysis

**Fig. 2:**
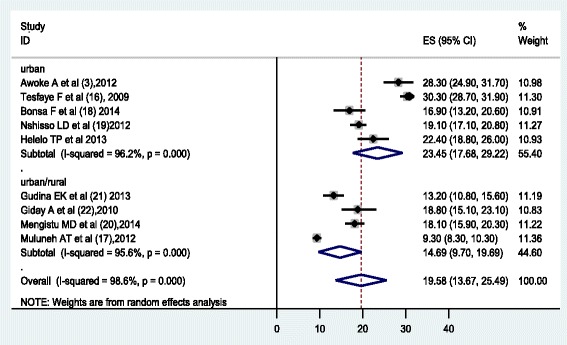
Forest Plot of the 9 studies that quantitatively assessed prevalence of hypertension in the Ethiopian by settings (urban/urban & rural), 2014

**Fig. 3:**
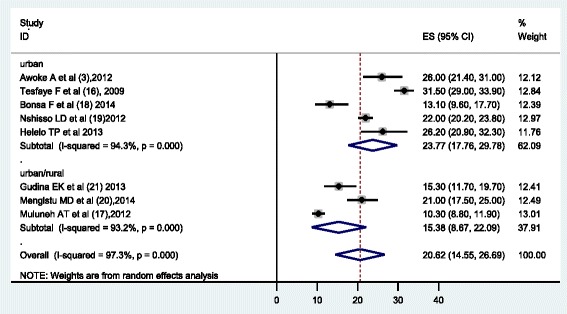
Forest Plot of the 8 studies that quantitatively assessed the prevalence rate of hypertension among males in Ethiopia by residence, 2014

**Fig. 4:**
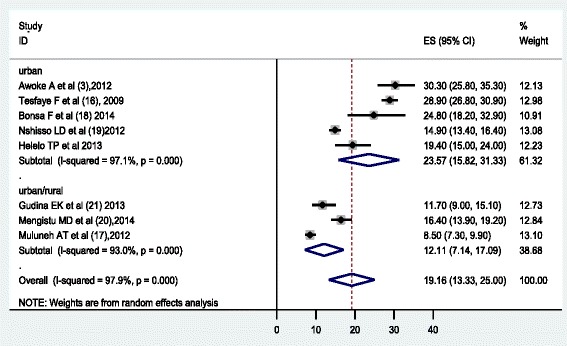
Forest Plot of the 8 studies that quantitatively assessed the prevalence rate of hypertension among females in Ethiopia by residence, 2014

The analysis of nine studies, according to the Der Simonian-Laird random-effects model, revealed that the pooled prevalence of hypertension among the Ethiopian population was 19.6 % (95 % CI: 13.7 %, 25.5 %) (Fig. 2). Subgroup analyses showed that the prevalence of hypertension in the urban population was 23.5 % (95 % CI 17.7, 29.2) and that of both rural & urban population was 14.7 % (95 % CI: 9.7, 19.7) (Fig. 2). This meta-analysis also revealed that prevalence of hypertension among males and females was 20.6 % (95 % CI: 14.6 %, 26.7) and 19.2 (95 % CI: 13.3 %, 25.0) respectively, which was estimated by using eight studies (Figs. 3 & 4).

## Discussion

This meta-analysis determined the pooled prevalence of hypertension in Ethiopia using nine studies. According to the results of this meta-analysis, the prevalence of hypertension in Ethiopia was estimated to be 19.6 % (23.5 % in urban population and 14.7 % in rural/urban population). This study also shown that the prevalence of hypertension in males and females was 20.6 % and 19.2 % respectively.

The highest prevalence of hypertension (30.2 %) was reported in a study done in Addis Ababa urban population [16]. But , Muluneh et al. detected a lower prevalence of hypertension (9.3 %) in urban/rural population of South West Ethiopia (Gilgel Gibe) [17]. The overall pooled prevalence in our study is comparable to a nationwide study in Gambian population [24]. But, it is lower than a community-based study conducted in one Uganda district [25]. This dissimilarity might be due to difference in study subjects. For example our analysis considers adults older than 15 years of age but the Ugandan study included study subjects who are 20 years and older. There might be also socio-economic and cultural differences.

The prevalence of hypertension was found to be higher in the urban population than the mixed urban/rural population one. This is consistence with the results of a systemic review in Africa and sub-Saharan Africa [26, 27]. The higher prevalence of hypertension in urban areas might be due to living lifestyle differences. Higher levels of obesity, increased salt and fat intake from consuming more processed foods and engaging in jobs with minimal physical activity are possible explanations for higher hypertension in urban populations

Awoke et al. (28.3 %) [3], Tesfaye et al. (30.27 %) [16], Bonsa et al. (16.9 %) [18] and Nshisso et al. (19.1 %) [19] reported that the prevalence of hypertension in urban population ranged 16.9 % - 30.27 % in between 2009 and 2014. This meta-analysis summarized all these findings and shows the prevalence of hypertension was 23.5 % in the urban population of Ethiopia.

In the combined rural/urban population, the highest prevalence of hypertension (18.8 %) was reported in Sidama [23] and the lowest prevalence (9.3 %)) was observed in the South West Ethiopia (Gilgel Gibe) [17]. This meta-analysis shown a pooled prevalence rate of 14.7 % among the rural/urban population of Ethiopia. This is higher than the result of study done in Rural Nigeria. The difference might be due to sociocultural factors and study periods difference, as the Nigeria study was conducted in 1991–1995 [26].

The lower prevalence among males (10.3 %) and females (8.5 %) was reported by Muluneh et al. [17] while the higher prevalence, 31.5 % among males and 30.3 % among females was documented by Tesfaye et al. [16] and Awoke et al. [3] respectively. This meta-analysis found the overall pooled prevalence of hypertension was 20.6 % among males and 19.2 % among females. This is inconsistence with study done in Gambia [24], which reported higher prevalence among females. This difference might be due to socio cultural difference and different criteria used to diagnose hypertension, the Gambia study used SBP/DBP > =160/95.

Even though hypertension is easily diagnosable and treatable with lifestyle modifications and effective medicines and it was 7^th^ contributor to premature death in developing countries [7] and responsible for 12 % of the CVD deaths in Addis Abba, Ethiopia [11]. Furthermore, hypertension control provides an entry point to deal with other NCDs as any intervention will help to concomitantly address other NCDs as well [6, 9]. Health education programs that promote exercise, weight reduction, early diagnosis and screening are some of the key interventions that will be promoted at various levels of health facilities. Ethiopia has been effectively implementing the health extension program by deploying health extension workers at grass root level in the community for the maternal and communicable diseases. This program should also incorporates hypertension as one component and health extension workers can carried out screening and teach the community about prevention mechanisms.

### Strengths and limitations

The strength of this study includes use of multiple databases to search articles (both manual and electronic search) for meta-analysis, abstraction of information uniformly using a predetermined and pretested standard format by two independent reviewers that helped to minimize error. This meta-analysis also included studies from different parts of the country that comprises both urban and rural population.

There are some potential limitations to this study. This analysis was based on limited studies (only nine cross sectional studies) which have had varied study subjects in terms of age, socio-cultural and bio-behavioral characteristics that might have effect on prevalence of hypertension. Even though it incorporates articles from different regions of the country, still the representativeness of the population is not as strong. We used the same criteria for the diagnosis of hypertension (systolic blood pressure ≥140 mmHg and/or diastolic blood pressure ≥ 90 mmHg or self-reported use of antihypertensive medication) for all articles included in the analysis. But still diagnosis of hypertension might be varied between studies and could have an impact on the result.

The analysis was based on studies having different characteristics. To address the issue of potential variability across studies, the analysis was performed by using random effect model. Under the random effects model the true effects in the studies are assumed to vary between studies and the summary effect is the weighted average of the effects reported in the different studies. The random effect model takes into considerations of any heterogeneity inherent in the meta-analysis and tend to give more conservative estimate.

## Conclusion

The pooled estimate does provide an overview of the magnitude of the problem of hypertension in the Ethiopian population. This evidence suggests Ethiopia is affected by double burden diseases so that policies and interventions should give attention and prioritization for reduction of hypertension in the Ethiopian adult population. A rising prevalence of hypertension in the population must trigger the policy makers and health care professionals as this is an area where primary prevention measures can bring about a substantial reduction in cardiovascular morbidity and mortality in the future. Further national population based studies are required for more accurate estimate of the prevalence of hypertension in the urban and rural population of the Ethiopia.

## Abbreviations

CVD: Cardiovascular Diseases
DALYS: Disability Adjusted Life Years
DBP: Diastolic Blood Pressure
NCDs: Non Communicable diseases
SBP: Systolic Blood Pressure
WHO: World Health Organization
